# CFD based design optimization of dimples induced on Blended Wing Body airframe using the Taguchi method

**DOI:** 10.1371/journal.pone.0320885

**Published:** 2025-04-17

**Authors:** Haris Ali, Mohammad Rasidi Rasani, Zambri Harun, Muhammad Ashhad Shahid

**Affiliations:** 1 Department of Mechanical and Manufacturing Engineering, Faculty of Engineering and Built Environment, Universiti Kebangsaan Malaysia (UKM), Bangi, Selangor, Malaysia; 2 Department of Mathematical Sciences, Faculty of Science and Technology, Universiti Kebangsaan Malaysia (UKM), Bangi, Selangor, Malaysia; Gazi University: Gazi Universitesi, TÜRKIYE

## Abstract

This research focuses on optimizing the design of dimples on a Blended-Wing-Body (BWB) airframe to enhance aerodynamic efficiency. Dimples serve as a passive flow control method intended to improve aerodynamic properties. Employing the Design of Experiments (DOE) framework and utilizing the Taguchi method, we examined five dimple design variables across three distinct levels. These variables included dimple placement, indentation depth, diameter, spacing between dimples, and the number of dimple rows on the BWB wing. An L_18_ orthogonal array (OA) was implemented to assess the impact of these variables on the drag coefficient (*C*_*D*_), lift coefficient (*C*_*L*_), and lift-to-drag ratio (*L/D*), which were used as performance metrics. High-fidelity Computational Fluid Dynamics (CFD) simulations were conducted for each of the eighteen configurations outlined by the L_18_ OA, across angles of attack ranging from 0° to 8°. Signal-to-Noise Ratio (SNR) analysis and Pareto Analysis of Variance (ANOVA) revealed that the dimple diameter had the most significant impact on both *C*_*D*_ and *L/D*, contributing 35.19% and 40%, respectively, while the indentation depth showed the least influence. The study identified an optimal combination of design variables (A_1_B_1_C_1_D_3_E_3_), which minimizes *C*_*D*_ and maximizes *L/D*. This work provides actionable guidelines for dimple design as a passive flow control method in aerospace applications.

## 1. Introduction

The Blended-Wing-Body (BWB) aircraft, featuring a seamless integration of high-lift wings with a broad, airfoil-shaped fuselage, has recently attracted significant interest in contemporary aviation due to its enhanced aerodynamic performance and lower fuel or energy consumption. These benefits stem from the design’s minimized wetted area and reduced overall weight [1]. Additionally, the BWB configuration offers a substantial increase in internal volume, accommodating the placement of larger and heavier payloads. Liebeck first introduced the concept of the BWB for high-speed subsonic commercial aircraft [2]. Subsequent to this seminal proposal, numerous researchers have explored the potential of this configuration for various applications, including commercial airliners [3,4], Unmanned Air Vehicle (UAV) utilization [5–7], and cargo transport [8]. Several studies have highlighted an enhancement of up to 20% in the *L/D* ratio of BWB configurations, predominantly attributed to the absence of empennage [9,10].

However, despite advancements, the imperative to enhance its aerodynamic efficiency, particularly in drag reduction and improved *L/D* ratio, persists, necessitating innovative solutions for improved efficiency and sustainability in modern aviation. For civil and commercial transport aircraft, irrespective of size, skin friction drag usually accounts for about 40–50% of the total drag encountered during cruise flight [11]. As such, even small reductions in drag can lead to significant advantages. Generally, the aerodynamic performance of BWB airframes can be enhanced by employing surface flow control methods, which are instrumental in reducing skin friction drag [12]. These methods are divided into two categories: active and passive. Passive strategies are often preferred over active ones because of their simplicity and cost-effectiveness, making them more practical for enhancing the aerodynamic efficiency of lifting surfaces. These methods influence stall phenomena and delay flow separation without relying on external energy sources.

One innovative passive flow control method is the introduction of dimples on the surface of interest. Dimples, are small concavities impressed upon a surface which act as a type of surface roughness, facilitate the development of a turbulent boundary layer, thereby delaying airflow separation, diminishing wake region, and reducing friction drag [13]. They have been subjected to thorough investigation in past owing to their ability to augment surface heat transfer [14,15]. Using dimples on bluff objects, such as spherical surfaces like golf balls, is well-recognized for its impact on the boundary layer turbulence and flow separation [16]. Asai *et al*. [17] carried out wind tunnel experiments to investigate volleyballs with dimpled and honeycomb-patterned surfaces. The findings showed a slight reduction in the critical Reynolds number for the modified balls. Additionally, the honeycomb-patterned balls exhibited a higher *C*_*D*_ compared to traditional designs, while dimpled balls demonstrated increased variability in flight orientation.

Many researchers also explored the impact of dimples in the context of automobile industry. Wang *et al*. [18] employed computational modeling to reduce drag on an automotive structure by incorporating a dimpled, textured surface. This method effectively modified the *C*_*D*_ by lowering turbulent kinetic energy and reducing wake vortex formation. Similarly, Chear and Dol [19] performed numerical calculations to investigate the influence of varying dimple depth and diameter combinations on the *C*_*D*_ of a car model. Their findings showed that all examined combinations contributed to drag reduction, with the maximum decrease in the *C*_*D*_ reaching 1.95%. Incorporation of dimples into streamlined objects like airfoils also have the potential to minimize wake size and postpone flow separation but studies are limited in context of aerospace and aviation industry. Allarton *et al*. [20] explored the use dimples on a NACA-6615 wing to enhance aerodynamic performance for automotive racing cars. Through CFD simulations and wind tunnel experiments, they investigate different dimple sizes at different angles of attack (AOAs). Their findings showed that the smallest dimples significantly improve performance at high AOA, reducing *C*_*D*_ and increasing downforce, thus enhancing traction and cornering in motorsport applications. Joseph *et al*. [21] carried out numerical studies at different AOAs to examine the effects of square dimples placed on the suction surface of a wing made up of NACA-0012 airfoil. Their findings indicated that these dimples might potentially prolong the stall angle by delaying the onset of stall.

Recently, many researchers investigated the influence of dimples to augment the aerodynamic efficiency of wind turbines. Azlan *et al*. [22] explored the impact of dimples on the aerodynamic performance of the horizontal axis wind turbine (HAWT) using CFD. Dimpled surfaces on the turbine blade’s suction side were found to increase output torque by up to 8.41%. The findings suggest that incorporating dimples can effectively improve the efficiency of wind turbines. In another study, Sedighi *et al*. [23] analyzed the effects of dimple radius and location on HAWT performance through numerical simulations. The study demonstrated a remarkable 16.08% improvement in torque, thereby highlighting the potential effectiveness of this passive modification in optimizing wind turbine performance.

Hence, in addition to bluff bodies, the incorporation of dimples into streamlined objects like turbine blades and aircraft wings also have the potential to minimize wake size and postpone flow separation by promoting turbulent boundary layer and reducing friction drag, but studies are limited in context of aerospace and aviation industry [24]. Moreover, the multitude of dimple design variables awaiting examination presents a formidable challenge, as aerodynamic studies are predominantly conducted using CFD. While CFD provides high-accuracy aerodynamic evaluations, it is often resource-intensive and time-consuming.

To optimize dimple design while minimizing computational demands, one efficient approach is to limit the design space by focusing on a limited set of potential parameter combinations. This approach helps reduce the time and resources needed for trade research and optimization. Nevertheless, it is essential to ensure that this reduction in design scope does not compromise the accuracy of the analysis or the quality of the optimized results. The Design of Experiments (DOE) technique is a potential way to balance these issues. DOE applies statistical techniques to define the design space and strategically allocate resources before conducting an “experiment.” In this context, “experiment” refers to any optimization or trade study, whether conducted experimentally or via computational simulations.

The DOE is widely utilized in various aeronautical research projects, including the multifaceted design optimization of airframe wing structures, as demonstrated in [25]. In this particular case, DOE is applied to establish the design space for a Surrogate-Based Optimization (SBO) process that incorporates CFD analysis. Similarly, in [26], DOE is utilized to explore the influence of multiple design parameters, assisting in solving a lightweight optimization challenge for a variable-span, shape-shifting wing.

Among the various DOE methods, Taguchi’s approach stands out as one of the most widely adopted and efficient [27,28]. It simplifies experimental design, result analysis, and experimentation time by addressing the challenges of both full and fractional factorial experiments. Taguchi introduces a specific set of Orthogonal Arrays (OAs) that minimize the complexity of fractional factorial designs, significantly streamlining the process. This method determines the optimal combination of design parameters, or control factors, for each performance metric. It evaluates the impact of all design variables on performance metrics while reducing the required time and resources. This efficiency allows for the integration of more costly experimental techniques or high-fidelity computational tools. Additionally, the method incorporates the use of a signal-to-noise ratio (SNR) and analysis of variance (ANOVA) to examine and interpret the results effectively.

Given its practical nature, numerous Taguchi-based optimization studies can be found across various branches of mechanical engineering. For instance, in manufacturing engineering, studies like those in [29,30] examine the effects of machining parameters on material and process quality. In the aerospace field, the Taguchi method has been used in several studies to investigate the parametric influence of canards on flying wings and leading-edge slats on BWB UAV configurations [31,32]. Additionally, this approach has been applied to determine the finest combination of aspect ratio, taper ratio, and sweep angle for the layout optimization of a tactical BWB platform, as demonstrated in [33]. Therefore, the Taguchi method holds promise as a candidate for conducting trade studies during the design optimization of dimples on BWB airframes. However, to the best of our knowledge, no research to date has employed the Taguchi method to explore the effects of dimple design variables on BWB wing configurations.

**Fig 1:**
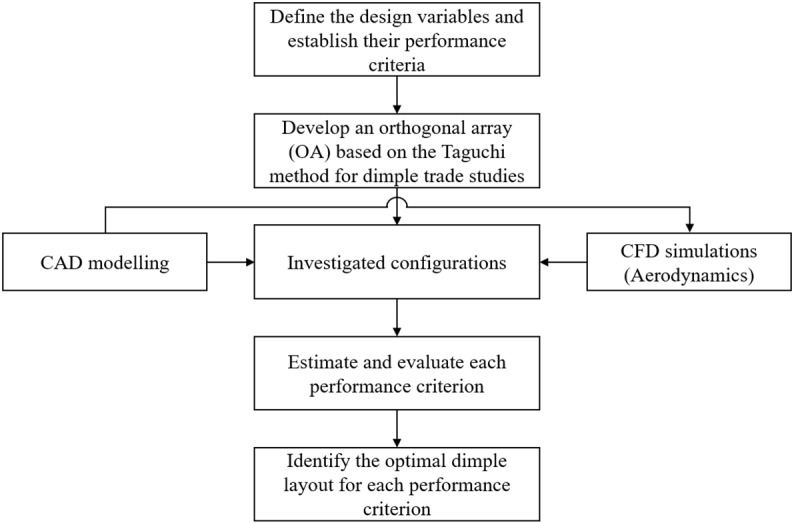
Optimization procedure for dimple design parameters using the Taguchi method.

In this study, a comprehensive parametric investigation of key design parameters of dimples integrated on the wings of BWB airframe is performed. The objectives of this study are to provide trends for the design of dimples as a passive flow control method for aerospace applications and also to identify critical design parameters of dimples which will influence the aerodynamic performance of BWB airframe. CFD approach is employed for the aerodynamic analysis combined with DOE framework to reduce analysis time, while preserving the predictions quality. By employing the Taguchi methodology, five dimple design variables are investigated on three distinct levels. The dimple placement location (*x/c*), indentation depth (*d*_*id*_), diameter (*D*_*d*_), spacing between dimples (*P*_*d*_), and the number of dimple rows (*r*_*d*_) on the BWB wing are selected as the investigated design variables, whereas the drag coefficient (*C*_*D*_), lift coefficient (*C*_*L*_), and lift-to-drag ratio (*L/D*) are selected as the performance criteria. Additionally, Pareto ANOVA is applied to determine the percentage share of each design variable to the optimized performance metrics. Fig 1 shows a comprehensive road map for the suggested optimization procedure.

## 2. Baseline BWB configuration

**Fig 2:**
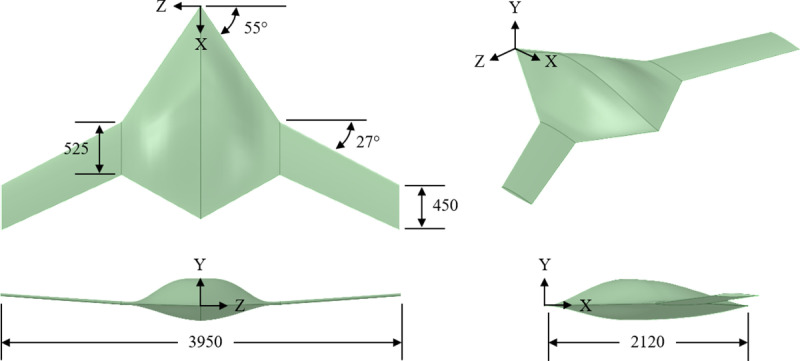
External layout of BWB airframe, all dimensions are in millimeters (mm).

The baseline configuration employed in this study adopts an optimized fixed-wing BWB airframe geometry [34]. Airfoil profiles were extracted at various spanwise locations, extending from the central body to the outer wing, inclusive of the wing tip. These profiles functioned as the foundational elements for generating all geometries investigated in this study. The root chord (*c*_*r*_), at the center of the body measures 2.12m, the tip chord (*c*_*t*_), at the outermost section of the wing is 0.45m, and the kink chord (*c*_*k*_), at the intersection of the body and wing is 0.525m. The body exhibits a sweep angle (*∧*_*b*_), of 55°, while the wing showcases a sweep angle (*∧*_*w*_), of 27°. Additional details about the design procedure, layout, and tools utilized in this study are available in [34]. The external layout of the reference BWB airframe, presented in Fig 2, serves as the benchmark configuration for the integration of dimples. The fundamental geometric details pertinent to the current investigation are presented in Table 1.

**Table 1:** Baseline BWB airframe specifications.

Parameter	Definition/value
Body sweep angle, *∧*_*b*_	55°
Wing sweep angle, *∧*_*w*_	27°
Body taper ratio, *λ*_*b*_	0.25
Wing taper ratio, *λ*_*w*_	0.85
Root chord, *c*_*r*_	2.12 m
Kink chord, *c*_*k*_	0.525 m
Tip chord, *c*_*t*_	0.45 m
Mean aerodyamic chord, *MAC*	0.91 m
Reference span, *b*_*ref*_	3.95 m
Reference area, *S*_*ref*_	3.50 m^2^

## 3. Tools and methods

This section provides a detailed description of the Taguchi technique and the associated methods employed for optimizing the dimple design on a BWB airframe. The process begins by defining the dimple design variables and their corresponding levels. Taguchi based OAs are then applied to generate the design space, which, in this study, results in 18 distinct BWB layouts with dimples for evaluation. For each configuration, a specific CAD model is created, followed by aerodynamic analysis through the solution of multiple CFD cases at different AOAs. Once the aerodynamic data is obtained, the optimal dimpled BWB configuration for each performance criterion is determined by SNR analysis. ANOVA is used in conjunction with this to assess the influence of the input design variables.

### 3.1. Parametric dimple design methodology

**Fig 3:**
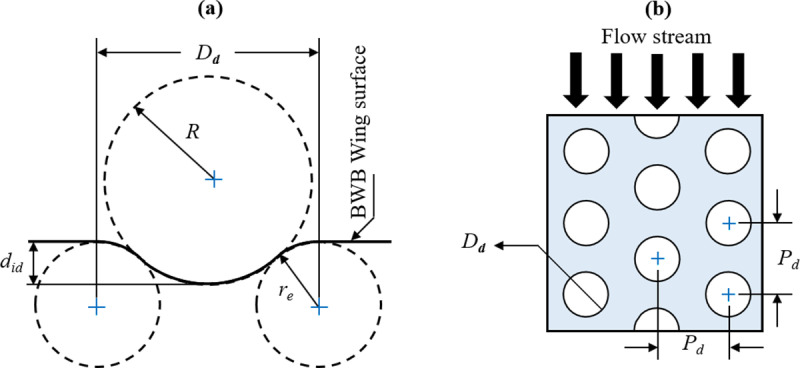
(a) Schematic of spherical dimple design (depth is exaggerated for illustration purpose); (b) Dimples layout.

A planar solid surface modified with dimples is characterized by various design variables. Emphasizing the significance of a precisely defined parametric geometry is crucial in the pursuit of achieving optimal dimple performance. Chen *et al*. [35] introduced a specific type of circular dimple, originally designed as spherical indentations with circular footprints, which has gained popularity for its parametric nature and is utilized in our current investigation. This dimple design arises from the combination of a spherical indentation and a torus, intersecting tangentially in a uniform manner to eliminate sharp edges. The dimples are arranged in a staggered pattern with a specific spacing (*P*_*d*_) between two adjacent dimples. This configuration has been reported to be more effective for flow control [13]. The cross-sectional view of the axially symmetrical dimple is shown in Fig 3a, while Fig 3b illustrates the staggered arrangement.

The geometric attributes of this dimple are defined by four parameters: the diameter of the circular dimple (*D*_*d*_), the indentation depth of the spherical ball (*d*_*id*_), the curvature radius at the intersection of wall and spherical ball (*r*_*e*_) and the curved radius of the spherical surface (*R*). The following analytical formula establishes the relationship among these dimple parameters [13]:


$$D_{d}=2\sqrt{d_{id}\left(2R+2r_{e}-d_{id}\right)}$$ (1)

The curved radius of the spherical surface was directly related to the diameter of the circular dimple and, therefore, was not treated as an independent variable in this study. The curvature radius at the intersection of wall and spherical ball was also not included as an independent variable to avoid too much complexity in the design procedure and kept constant as 0.5*D*_*d*_.

Consequently, the number of geometric parameters considered in the current trade study for dimple design was reduced from four to two, allowing to include other dimple design variables to be considered with respect to their integration on the wings of BWB airframe. The remaining variables to be included in the optimization studies are the dimple placement location (*x/c*) on the wing of BWB airframe, the spacing between adjacent dimples (*P*_*d*_) as shown in Fig 3b, and the number of dimple rows (*r*_*d*_) on the BWB wing. Hence, the OA of the Taguchi method was determined by five parameters: the placement location (A), the indentation depth (B), the dimple diameter (C), the spacing between dimples (D) and the number of rows (E).

To ensure clarity and consistency, all dimple dimensions are normalized using standard aerodynamic conventions. Specifically, the dimple diameter (*D*_*d*_) is normalized with respect to the kink chord (*c*_*k*_), while other parameters such as the indentation depth (*d*_*id*_) and spacing between dimples (*P*_*d*_) are normalized with respect to *D*_*d*_. Additionally, *r*_*d*_ is used to denote the number of dimple rows and *x/c* represents the normalized placement location of the dimples along the chord length. These notations are unique, descriptive, and consistently applied throughout the manuscript in equations, tables, and figures. A summary of all symbols is provided in Table 2 for quick reference.

**Table 2:** Notation and normalization conventions for dimple design parameters.

Symbol	Definition	Units
*D*_*d*_	Dimple diameter	Proportion of *c*_*k*_
*d*_*id*_	Indentation depth of dimples	Proportion of *D*_*d*_
*P*_*d*_	Spacing between adjacent dimples	Proportion of *D*_*d*_
*r*_*d*_	Number of dimple rows	Dimensionless
*x/c*	Placement location of dimples	Dimensionless

**Fig 4:**
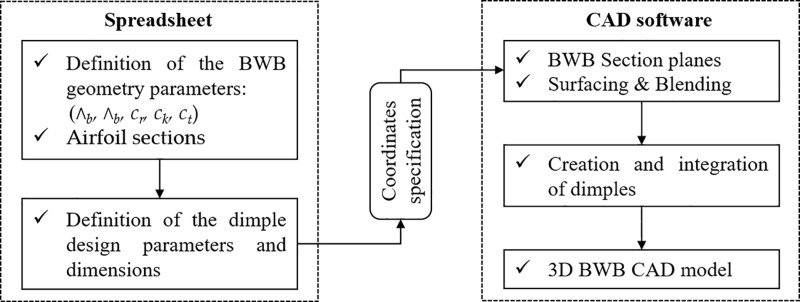
Methodology of the parametric CAD modeling.

**Fig 5:**
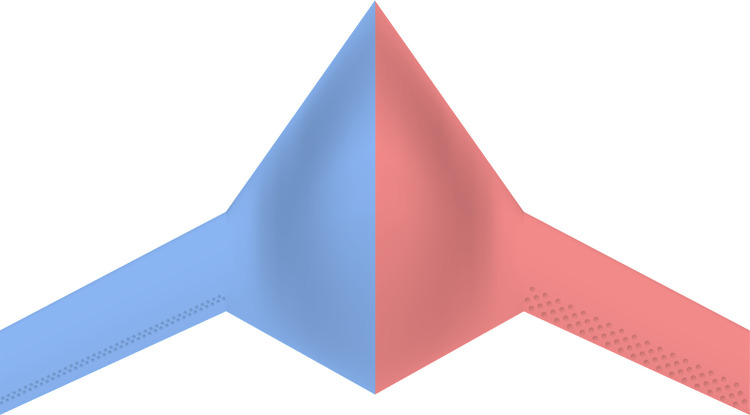
Indicative comparison of two dimple configurations from the L_18_ OA.

To perform the CFD simulations for each configuration derived from the L_18_ OA, a parametric 3D CAD model was implemented, ensuring accurate and consistent geometry across all configurations. The workflow for the design tool is illustrated in Fig 4. Commercial 3D CAD software was utilized to generate the various BWB configurations based on the selected dimple design parameters. The BWB’s main body was first created, followed by the addition of dimples on the wings as separate solid bodies. Finally, the dimples were subtracted from the main wing structure to produce the desired configurations. Both wings of the BWB airframe were modified by integrating dimples near the trailing edge (TE) at 85% of *c*_*k*_. No dimples were applied to the fuselage or body of the airframe. Fig 5 showcases the top view of two dimple configurations from the L_18_ OA, demonstrating the CAD modeling process. Configuration 7 appears in blue on the left, while configuration 3 is depicted in red on the right.

### 3.2. Taguchi methodology

In modern aviation, advanced computational techniques and experimental methods are widely employed. While these approaches enable crucial optimization and trade-off studies, they are often limited by the significant demands they place on processing time, resource allocation, and manual input. As a result, the scope of the design space is frequently restricted, as illustrated by some studies about the winglet optimization reported in [36]. The complexity and expense of these studies increase even more when low-fidelity approaches, like vortex lattice methods, fail to accurately model intricate 3D configurations, such as the aerodynamic effects of dimples on a BWB airframe.

The total number *N* of possible combinations for *L* levels of *m* design variables in a full factorial optimization study, where the entire design space is considered, is given by the following expression:


$$N=L^{m}$$ (2)

To minimize the resource and time demands of a full factorial analysis while still exploring the complete design space, Taguchi proposed a standardized DOE technique [37]. This approach, commonly adopted in the industry, enhances the creation of top-notch products by maximizing their durability and minimizing their vulnerability to unpredictable elements, all while keeping costs relatively low. Taguchi’s method accomplishes this by utilizing a specific set of OAs, which outline the minimum number of desired “tests” for the selected design variables, as outlined in Table 3. These tests guarantee that the conclusions derived from the smaller test set are still applicable for the whole design space.

**Table 3:** Comparative illustration between full factorial and Taguchi experimental designs.

Factors	Levels	Total number of experiments	
		Full factorial design	Taguchi design
2	2	2^2^ = 4	4
3	2	2^3^ = 8	4
4	2	2^4^ = 16	8
15	2	2^15^ = 32768	16
4	3	3^4^ = 81	9
5	3	3^5^ = 243	18
6	3	3^6^ = 729	27

While the Taguchi method is a well-known and widely applied DOE approach across various industries, the novelty of this study lies in its integration of Taguchi with ANOVA to quantify the impact of each dimple design parameter on the aerodynamics of the BWB airframe. This distinctive combination has not been explored in prior aerospace research.

The process of developing OA begins with determining the total degrees of freedom (DOF), which determines the least number of “tests” needed to evaluate the selected design variables. The DOF for each design parameter are determined by subtracting one from the number of levels (Equations (3–7)). Therefore, the total DOF are calculated by summing the DOF for each design parameter’s main effect, along with an additional DOF related to the overall mean [38].


$$df_{A}=numberoflevelsofparameterA-1$$ (3)

$$df_{B}=numberoflevelsofparameterB-1$$ (4)

$$df_{C}=numberoflevelsofparameterC-1$$ (5)

$$df_{D}=numberoflevelsofparameterD-1$$ (6)

$$df_{E}=numberoflevelsofparameterE-1$$ (7)

The subsequent step involves selecting the appropriate layout for the OA, which is dictated by the total DOF and the quantity of design variables. The number of columns in the OA corresponds to the factors and their respective levels, while the number of rows must be no less than the total DOF. Field experts choose the design parameters based on the relevant field, theoretical considerations, and industry best practices. In this study, the design variables described earlier are provided in Table 4. Specifically, the selected design parameters include the dimple placement location, *x/c* (A), indentation depth, *d*_*id*_ (B), dimple diameter, *D*_*d*_ (C), spacing between dimples, *P*_*d*_ (D), and the number of dimple rows, *r*_*d*_ (E). These five parameters are considered the most critical in the design and integration of dimples on BWB airframe wings, as they significantly impact the aerodynamic efficiency and, consequently, the overall performance of the airframe.

**Table 4:** Investigated design parameters and their corresponding levels.

Parameters	Levels		
	I	II	III
A, *x/c* [0.85*c*_*k*_]	Suction side (SS)	Pressure side (PS)	–
B, *d*_*id*_ [% *D*_*d*_]	0.025	0.05	0.075
C, *D*_*d*_ [% *c*_*k*_]	0.01	0.025	0.04
D, *P*_*d*_ [% *D*_*d*_]	1.5	2.5	3.5
E, *r*_*d*_ [-]	2	3	4

The values for each level of design parameter were chosen on the basis of a reference BWB airframe, manufacturability constraints, and general guidelines for dimple design [13,39]. As a result, the arrangement of design variables and their respective levels (one parameter with two levels and the remaining four parameters with three levels each) produced an L_18_ OA for the present optimization study, as shown in Table 5. Eighteen distinct dimple layouts were analyzed, and their impact on the aerodynamic characteristics of the BWB airframe was assessed. It is important to highlight that, through the use of the Taguchi method, the design space was significantly reduced from 243 possible configurations to just 18. This significant decrease in the number of layouts greatly minimized the CAD and CFD analysis workload compared to a full factorial approach.

**Table 5:** L_18_ Orthogonal Array.

Configuration	Parameters				
	A	B	C	D	E
1	SS	0.025	0.01	1.5	2
2	SS	0.025	0.025	2.5	3
3	SS	0.025	0.04	3.5	4
4	SS	0.05	0.01	1.5	3
5	SS	0.05	0.025	2.5	4
6	SS	0.05	0.04	3.5	2
7	SS	0.075	0.01	2.5	2
8	SS	0.075	0.025	3.5	3
9	SS	0.075	0.04	1.5	4
10	PS	0.025	0.01	3.5	4
11	PS	0.025	0.025	1.5	2
12	PS	0.025	0.04	2.5	3
13	PS	0.05	0.01	2.5	4
14	PS	0.05	0.025	3.5	2
15	PS	0.05	0.04	1.5	3
16	PS	0.075	0.01	3.5	3
17	PS	0.075	0.025	1.5	4
18	PS	0.075	0.04	2.5	2

### 3.3. CFD methodology

The aerodynamic performance of each layout from the L_18_ OA was evaluated using high-fidelity CFD simulations. This study employs steady-state simulations to compare aerodynamic coefficients across various dimple configurations, focusing on subsonic cruise conditions and moderate AOAs (0°–8°). The primary objective was to establish design trends and optimize dimple configurations, rather than to investigate transient flow dynamics. The steady-state approach was chosen for its computational efficiency and ability to identify key aerodynamic trends with sufficient accuracy for the intended scope. While we acknowledge that unsteady effects, particularly at higher AOAs, may influence aerodynamic behavior, an in-depth examination of near- and post-stall phenomena is beyond the scope of this study. Future research could extend this analysis by incorporating unsteady simulation methods, such as URANS or LES, to better capture dynamic flow characteristics at higher AOAs and evaluate the role of dimples under those conditions.

The Reynolds-Averaged Navier-Stokes (RANS) equations are solved, together with the *k-ω* SST turbulence model, developed by Menter [40], for resolving the flow field. The *k-ω* SST turbulence model was selected due to its well-documented ability to accurately predict flow behavior in scenarios involving adverse pressure gradients and flow separation, which are critical for assessing the aerodynamic performance of dimpled surfaces. This model combines the strengths of the *k−ω* model in the near-wall region with the *k-ε* model’s robustness in the free-stream region, providing a balanced approach for high-fidelity simulations of complex aerodynamic flows. Numerical simulations were performed using ANSYS FLUENT to solve the RANS equations. The specific solution methods used in these simulations are outlined in Table 6.

**Table 6:** CFD solution method.

Parameter	Definition
Pressure and velocity	Coupled
Gradient	Least squares cell-based
Pressure and momentum	Upwind - 2^nd^ order
Turbulent kinetic energy, *k*	Upwind - 2^nd^ order
Specific dissipation rate, ω	Upwind - 2^nd^ order

The CAD models were imported into the Fluent mesher to discretize the surrounding control volume and generate the appropriate computational meshes. Since the BWB airframe exhibits symmetry, only half of the model was simulated to conserve computational resources and time. In this study, both structured and unstructured meshes were employed to resolve the flow field. A structured mesh was applied near the surface of the BWB airframe to accurately capture the flow and its sharp gradients within the boundary layer, while the remainder of the domain was discretized with an unstructured mesh for greater flexibility and reduced mesh size. A high grid point density was maintained near the dimples and around the leading and trailing edges of the BWB airframe to ensure precise representation of the flow dynamics.

**Fig 6:**
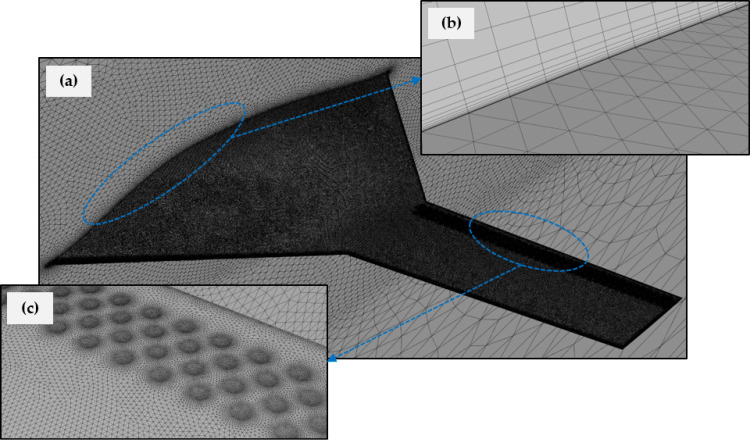
Generated mesh; (a) In domain and BWB; (b) Inflation layers; (c) Fine resolution near dimples.

It is important to keep the y + parameter under 3 to ensure precise representation of the boundary layer in turbulent flow simulations when using the *k-ω* SST turbulence model [41]. To accomplish this, twenty-six prism layers were added adjacent to the walls, with the initial layer height set at 1.5 ×  10^-5^ meters and a growth rate of 1.24. This configuration results in a y + value of approximately 1, ensuring that the near-wall mesh is sufficiently refined to capture the details of the viscous sublayer. Fig 6 displays the entire computational domain along with a detailed view of the mesh in the dimpled region and inflation layers.

**Fig 7:**
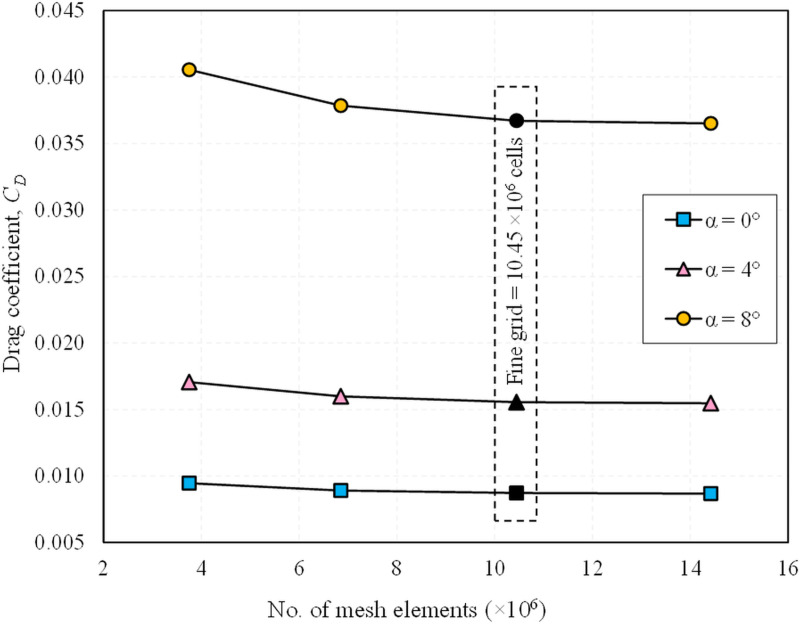
Grid independence study (drag coefficient, *C*_*D*_) performed on configuration 4 of the L_18_ OA.

A comprehensive grid independence study was performed to ensure that grid resolution did not influence the accuracy of the results. Four different mesh resolutions—coarse, medium, fine and very fine—were generated, comprising 3.75 million, 6.85 million, 10.45 million and 14.42 million cells, respectively. The configuration 4 of the L_18_ OA was selected for this study and the finalized mesh settings were implemented for the remaining configurations. The *C*_*D*_ was used as the metric for evaluating grid independence. Fig 7 presents the variations in *C*_*D*_ for the different mesh resolutions at various AOAs. The results showed a deviation of approximately 6% to 7% between the coarse and medium meshes, while the deviation between the medium and fine meshes was approximately 2.3% to 3% at various AOAs. However, the variation in *C*_*D*_ for the fine and very fines meshes was less than 0.56%, even at the highest AOA of 8°. Consequently, the fine mesh comprising of 10.45 million cells was selected for subsequent simulations to balance computational efficiency and accuracy.

The simulated configurations were analyzed over a broad range of AOAs, spanning from 0° to 8°, under sea level conditions. The selected range of AOAs was chosen to encompass the typical operational envelope of a BWB airframe during subsonic cruise and moderate maneuvering conditions. These angles represent the aerodynamic regime where such configurations are expected to operate efficiently, with minimal flow separation. This range allows us to focus on identifying design trends and optimal configurations under realistic and relevant conditions without delving into extreme angles that may result in significant flow separation or stall, which are outside the scope of this study. Table 7 provides a summary of the boundary and initial conditions, including the turbulence boundary parameters at the inlet [42].

**Table 7:** Boundary conditions employed in current study.

Parameter	Condition/Value
Flow-in	Velocity inlet
Flow-out	Pressure outlet
Freestream velocity	50 m/s
Altitude	0 m (Sea level)
Ambient temperature	288 K
Ambient pressure	101325 Pa
Turbulence intensity	0.01

### 3.4. SNR and ANOVA methodology

Taguchi introduced SNR as a key metric for analyzing results. Acting as both an efficiency gauge and a quality indicator connected to the loss function, SNR helps reduce process losses by being maximized. “Experimental/simulations” data is converted into SNR values using three distinct formulas, each tailored to the specific outcome being targeted (Equations (8), (9), (10)). The logarithmic transformation applied to observations in the SNR enhances the ability to predict improvements in the performance criteria.


$$Smallerthebetter:SNR=-10\log_{10}\overset{n}{\underset{1}{\sum }}y^{2}$$ (8)

$$Nominalthebetter:SNR=-10\log_{10}\overset{n}{\underset{1}{\sum }}\frac{\bar{Y}}{S_{y}^{2}}$$ (9)

$$Biggerthebetter:SNR=-10\log_{10}\overset{n}{\underset{1}{\sum }}\frac{1}{y^{2}}$$ (10)

In this instance, *y* represents the estimated performance metrics for each configuration, while *n* denotes the total layouts being analyzed. Additionally, $\bar{Y}$ refers to the mean value, and $S_{Y}^{2}$ indicates the variance for each performance metric (Equations (11), (12)) [43].


$$\bar{Y}=\frac{\sum_{1}^{n}y}{n}$$ (11)

$$S_{Y}^{2}=\frac{\sum_{1}^{n}{\left(y-\bar{Y}\right)}^{2}}{n}$$ (12)

In this study, the performance criteria were optimized using different SNR definitions. For minimizing *C*_*D*_, the ‘Smaller the Better’ criterion was employed, while for maximizing *L/D* ratio, the ‘Bigger the Better’ criterion was applied. The design parameter combination with the highest SNR for each performance metric is regarded as the most optimal, indicating superior performance with the least amount of variation.

Additionally, an ANOVA approach [37,43] was employed to further analyze the results and determine the optimal parameter combinations for each performance metric. ANOVA, a statistical method, is employed to evaluate “experimental/simulations” data and assess how design parameters influence overall variation in the results. This approach enables the identification of the statistical significance of each design variable in relation to the chosen performance criteria. In this study, ANOVA was applied to both the reduction in *C*_*D*_ and the increase in the *L/D* ratio, considering the SNR analysis results. This analysis provided insights into the relevance and proportional impact of input parameters *x/c*, *d*_*id*_, *D*_*d*_, *P*_*d*_ and *r*_*d*_.

According to the methodology described in [43], *i* corresponds to the number of design variables and *j* represents the levels for each variable, with *i* ranging from 1 to 5 and *j* ranging from 1 to 3 in this study (refer to Table 3). Similarly, *k* signifies the number of configurations, which in this case ranges from 1 to 18 (as shown in Table 4). The contribution of each design variable is determined by applying the standard ANOVA method, as outlined in Equation (13). This allows for a quantitative assessment of the influence each parameter has on the performance outcomes.


$$Percentagecontribution=\frac{SS_{i}}{SS}\times 100$$ (13)

The terms *SS*_*i*_ and *SS* represent the sum of squares of each design parameter and the total sum of squares, respectively, due to variation about the overall mean. The exact values of *SS*_*i*_ and *SS* are calculated using Equations (14) and (15).


$$SS_{i}=\overset{j}{\underset{1}{\sum }}{\left(SNR_{ij}-\bar{SNR}\right)}^{2}$$ (14)

$$SS=\overset{k}{\underset{1}{\sum }}{\left(SNR_{k}-\bar{SNR}\right)}^{2}$$ (15)

Lastly, *SNR*_*ij*_ represents the average SNR for the *i*^*th*^ parameter at the *j*^*th*^ level, while *SNR*_*k*_ corresponds to the SNR for the *k*^*th*^ configuration. The overall mean SNR is then determined using Equation (16), providing a baseline for evaluating the performance across all configurations.


$$\bar{SNR}=\frac{\sum_{1}^{k}SNR_{k}}{k}$$ (16)

This procedure is carried out for each performance criterion, resulting in five distinct percentage contributions in this study.

Additionally, an *F*-test is performed to determine the influence of studied design variables on the performance metrics. More precisely, the *F*-value for each variable is computed by dividing the mean square of the design variable by the mean square of the error (as shown in Equation (17)). This test helps assess whether the variation in the design parameter exert a meaningful statistical effect on the performance outcomes.


$$F_{i}=\frac{MS_{i}}{MS_{E}}$$ (17)

The mean squares of each design variable, *MS*_*i*_, and the mean squares of error, *MS*_*E*_, are in turn calculated using Equations (18) and (19):


$$MS_{i}=\frac{SS_{i}}{df_{i}}$$ (18)

$$MS_{E}=\frac{SS_{E}}{df_{E}}$$ (19)

In this context, *df*_*i*_ represents the DOF for the *i*^*th*^ variable, while *SS*_*E*_ and *df*_*E*_ refer to the sum of squares and DOF for the error, respectively. These values are determined using Equations (20) and (21), providing the necessary components for calculating the mean squares and conducting the F-test.


$$SS_{E}=SS-SS_{A}-SS_{B}-SS_{C}-SS_{D}-SS_{E}$$ (20)

$$df_{E}=df_{T}-df_{A}-df_{B}-df_{C}-df_{D}-df_{E}$$ (21)

Lastly, A, B, C, D and E refer to the dimple design variables mentioned in Table 3, and *df*_*T*_ represents the DOF, which is the number of layouts in the OA minus one. This process is carried out for each performance metric, producing five F-values. A higher F-value indicates a stronger influence of the associated design variable on the performance outcomes.

## 4. Results and discussion

The CFD analysis was segmented into multiple batches, where each batch represented the drag polar for a specific configuration. To define the drag polar, six different AOAs, i.e., − 2°, 0°, 2°, 4°, 6°, and 8°—were used for each configuration’s analysis. This resulted in a total of 108 CFD simulations using the Taguchi method, while a full factorial design would require 1,458 CFD cases. This approach reduced the number of “experiments” needed for the optimization trade studies by nearly 90%, significantly cutting down the analysis time.

**Fig 8:**
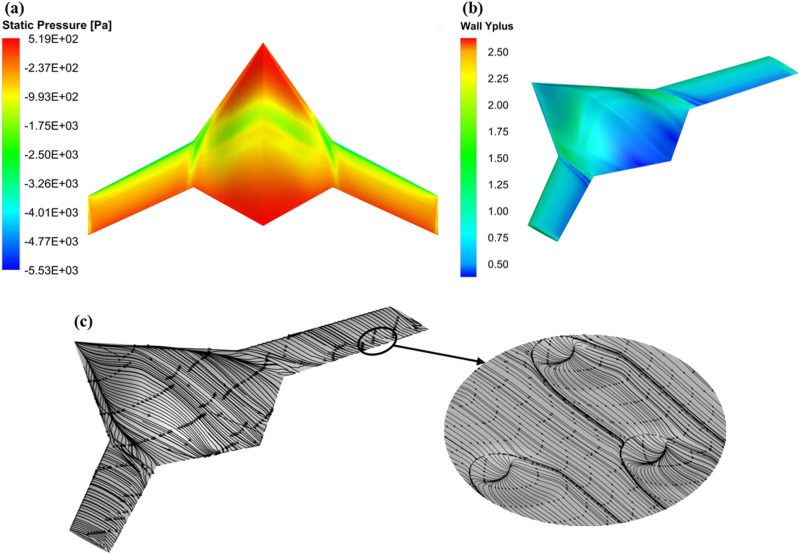
CFD analysis graphics for the BWB configuration 5 at **α =** **6°;** (a) Pressure distribution; (b) Wall y-plus (c); Flow streamlines.

**Fig 9:**
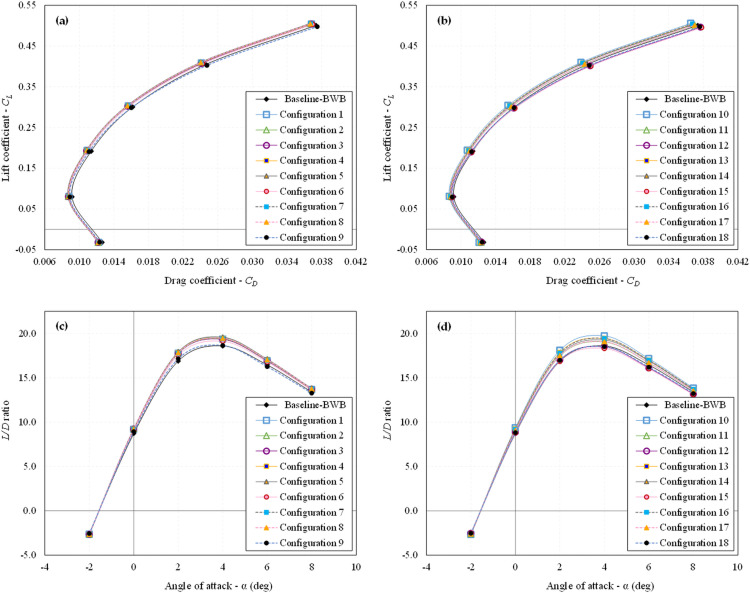
Aerodynamic characteristics of all dimpled BWB configurations; (a) Drag polar - SS; (b) Drag polar - PS; (c) L/D ratio – SS; (d) L/D ratio – PS.

Fig 8 illustrates the CFD results for configuration 5 indicatively. The first step involved extracting the results from the CFD simulations for all 18 configurations, providing key aerodynamic coefficients such as *C*_*L*_, *C*_*D*_, and *L/D* ratio. The impact of dimples on the aerodynamic properties of the BWB airframe is evaluated through drag polar and *L/D* ratio assessments. The variation in drag polar and *L/D* ratio for all the examined dimpled BWB configurations is presented in Fig 9a, 9b, 9c, and 9d, respectively.

After completing the numerical simulations, the optimal combination of design variables for each performance criterion was identified through SNR analysis. As outlined in Section 3.4, the performance criteria selected for optimization were the maximum reduction in *C*_*D*_ and the maximum enhancement of the *L/D* ratio. The SNR for each configuration, based on these performance criteria, is calculated and presented in Table 8. The analysis reveals that the mean SNR for the maximum reduction in *C*_*D*_ is 39.21 dB, while the mean SNR for the maximum improvement in the *L/D* ratio is 24.90 dB.

**Table 8:** Predicted performance criteria and computed SNR values.

Configuration	*C*_*D α = 2°*_	δ*C*_*D*_	SNR	*L/D*_*α = 2°*_	δ*L/D*	SNR
		(%)	(dB)		(%)	(dB)
1	0.01090	-4.08	39.2491	17.771	5.20	24.9941
2	0.01082	-4.80	39.3145	17.883	5.86	25.0490
3	0.01085	-4.53	39.2904	17.766	5.17	24.9920
4	0.01087	-4.34	39.2729	17.830	5.55	25.0229
5	0.01079	-5.06	39.3384	17.940	6.20	25.0765
6	0.01100	-3.20	39.1701	17.501	3.60	24.8614
7	0.01091	-4.01	39.2429	17.768	5.18	24.9929
8	0.01087	-4.33	39.2715	17.824	5.52	25.0203
9	0.01111	-2.26	39.0857	17.153	1.54	24.6867
10	0.01072	-5.69	39.3961	18.110	7.21	25.1583
11	0.01102	-3.02	39.1535	17.456	3.33	24.8387
12	0.01115	-1.86	39.0506	17.021	0.76	24.6196
13	0.01086	-4.46	39.2836	17.861	5.73	25.0381
14	0.01095	-3.65	39.2101	17.613	4.27	24.9168
15	0.01125	-1.07	38.9805	16.847	-0.27	24.5304
16	0.01089	-4.23	39.2623	17.783	5.27	25.0001
17	0.01099	-3.31	39.1795	17.512	3.66	24.8665
18	0.01121	-1.37	39.0074	16.987	0.56	24.6022

As discussed in Section 3.4, the “Smaller is Better” SNR criterion was adopted for optimizing maximum drag reduction, while the “Bigger is Better” criterion was utilized for maximizing the *L/D* ratio. The SNR analysis results for each individual variable are shown in Tables 9 and 10. The term “Delta” refers to the variation between the highest and lowest SNR values for each variable. The ranking of each variable, which indicates the degree of its impact on the response characteristic, is determined by dividing each variable’s delta by the sum of the deltas for all variables. This ranking reveals which variables have the most significant impact on the performance criteria.

**Table 9:** Response table for δ*C*_*D*_.

Level	A	B	C	D	E
1	39.25	39.24	39.28	39.15	39.17
2	39.17	39.21	39.24	39.21	39.19
3		39.17	39.1	39.27	39.26
Delta_max_	0.08	0.07	0.19	0.11	0.09
Rank	4	5	1	2	3
Contribution ratio (%)	14.81	12.96	35.19	20.37	16.67

**Table 10:** Response table for δ*L/D.*

Level	A	B	C	D	E
1	24.97	24.94	25.03	24.82	24.87
2	24.84	24.91	24.96	24.9	24.87
3		24.86	24.72	24.99	24.97
Delta_max_	0.13	0.08	0.32	0.17	0.1
Rank	3	5	1	2	4
Contribution ratio (%)	16.25	10.00	40.00	21.25	12.50

**Fig 10:**
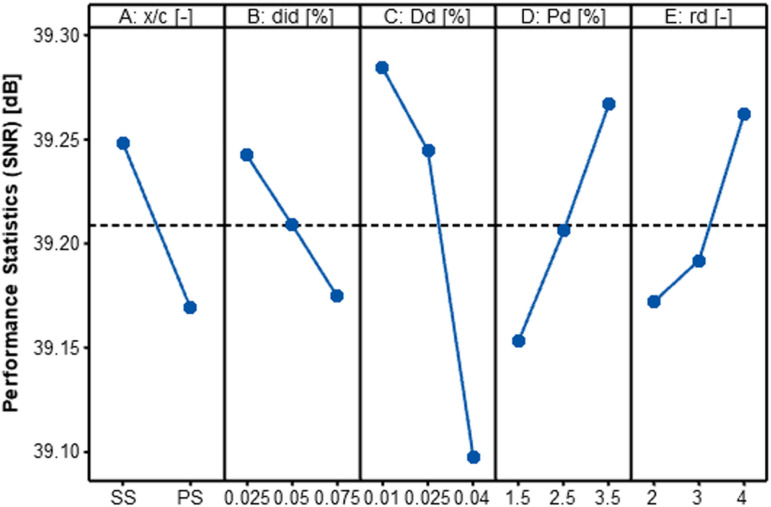
Impact of design variables on δ*C*_*D*_.

**Fig 11:**
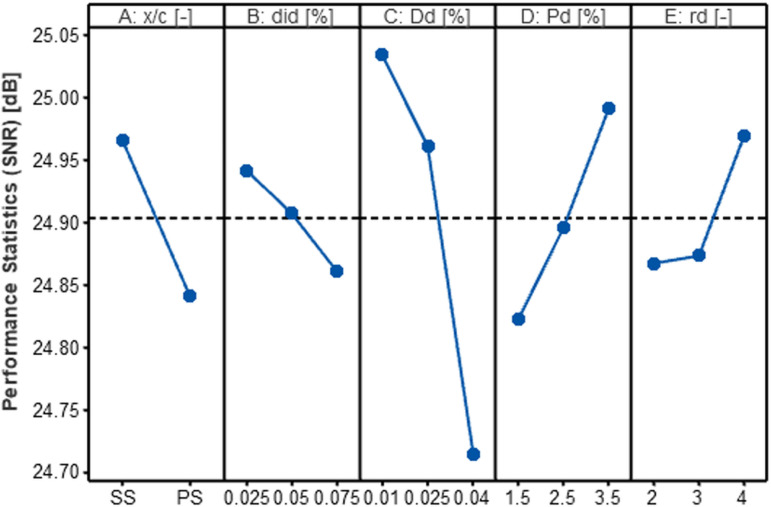
Impact of design variables on δ*L/D.*

The effect of the input variables on the evaluation metrics is depicted in Figs 10 and 11. The optimal level for each variable, based on each outcome characteristic, is determined by the highest SNR value. Both the drag reduction (δ*C*_*D*_) and the *L/D* ratio improvement (δ*L/D*) exhibited similar trends, reaching their maximum values at the level one for parameters A (*x/c*), B (*d*_*id*_), and C (*D*_*d*_), and at the level three for parameters D (*P*_*d*_) and E (*r*_*d*_).

Table 11 summarizes the optimal combinations of input variable levels for each performance metric examined. The combination A_1_B_1_C_1_D_3_E_3_ is identified as optimal for drag reduction, based on the “Smaller is Better” criterion and corresponding SNR analysis. Likewise, A_1_B_1_C_1_D_3_E_3_ is also the optimal combination for *L/D* ratio enhancement, determined by the “Bigger is Better” criterion of the SNR. In terms of dimple location, dimples placed near the trailing edge on the suction surface prove to be the most effective for both drag reduction and *L/D* ratio improvement. This greater effectiveness on the suction surface can be attributed to the stronger interaction with high-energy airflow, leading to improved boundary layer control and vortex generation.

**Table 11:** Optimal design variable combinations for the optimization of the performance metrics.

	Dimple design variables				
	A (*x/c*)	B (*d*_*id*_)	C (*D*_*d*_)	D (*P*_*d*_)	E (*r*_*d*_)
δ*C*_*D*_					
Optimum level	1	1	1	3	3
Optimum value	SS	0.025	0.01	3.5	4
δ*L/D*					
Optimum level	1	1	1	3	3
Optimum value	SS	0.025	0.01	3.5	4

For dimple depth, shallow dimples are found to be more effective than deeper ones. Across all optimized configurations, the smallest dimple diameter is preferred, suggesting that smaller dimples generate finer, more consistent vortices that enhance boundary layer mixing without causing excessive turbulence. In terms of dimple spacing, larger spacing yields the most significant aerodynamic benefits for the BWB platform, as increased spacing allows for efficient vortex generation and promotes boundary layer transition with minimal additional drag.

Finally, increasing the number of rows of dimples further enhances aerodynamic performance by increasing the overall number of dimples, which, in conjunction with other optimized dimple parameters, leads to improved aerodynamic efficiency of the BWB airframe.

The ANOVA results, displayed in Tables 12 and 13, highlight the impact of the design variables on each performance metric. As per the analysis, all parameters appear to be significant in reducing *C*_*D*_ and enhancing the *L/D* ratio with a P-value less than 0.05. *D*_*d*_ (C) is the variable with the highest impact on δ*C*_*D*_ and δ*L/D*. In contrast, *d*_*id*_ (B) has a less significant effect on δ*C*_*D*_ and becomes only marginally influential for δ*L/D*.

**Table 12:** ANOVA results for SNR related to δ*C*_*D*_.

Level	DOF	SS	MS	F	P (%)
A	1	0.028151	0.028151	62.48	12.39
B	2	0.013661	0.00683	15.16	6.01
C	2	0.116443	0.058222	129.22	51.24
D	2	0.038515	0.019258	42.74	16.95
E	2	0.026886	0.013443	29.84	11.83
Error	8	0.003605	0.000451		1.59
Total	17	0.227261			

**Table 13:** ANOVA results for SNR related to δ*L/D.*

Level	DOF	SS	MS	F	P (%)
A	1	0.07033	0.070331	52.65	12.55
B	2	0.01959	0.009793	7.33	3.49
C	2	0.33519	0.167593	125.47	59.80
D	2	0.08543	0.042714	31.98	15.24
E	2	0.03932	0.019659	14.72	7.01
Error	8	0.01069	0.001336		1.91
Total	17	0.56053			

**Fig 12:**
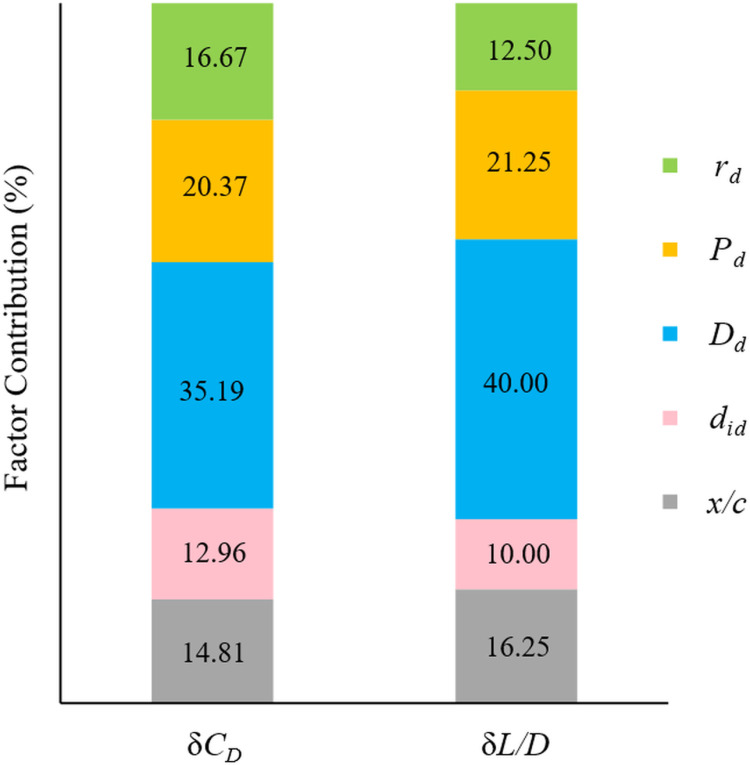
Individual share of design variables to each performance metric.

Lastly, Fig 12 illustrates the share of each design variable to the performance metrics through a bar chart representation. The authors emphasize that the insights drawn from Fig 12, along with the findings in Table 11, represent key outcomes of this optimization study. The chart provides a clear depiction of the influence of each design parameter. Notably, for both minimizing the *C*_*D*_ and maximizing the *L/D* ratio, *D*_*d*_ (C) emerges as the most influential factor, contributing 35.19% and 40%, respectively. In contrast, *d*_*id*_ (B) has the least impact, with contribution factors of 12.96% for drag reduction and 10% for improving the *L/D* ratio.

While this study demonstrates that *D*_*d*_ significantly influences the aerodynamic performance of the BWB airframe, its contribution might vary under different operating conditions. Specifically, at higher AOAs, where flow separation becomes more pronounced, the effectiveness of *D*_*d*_ in boundary layer control could differ. Additionally, under elevated turbulence intensities, the interaction between dimples and external turbulence structures may amplify or reduce their influence on drag and lift characteristics. Investigating these scenarios falls outside the scope of the current study but presents a compelling direction for future research to expand the applicability of the findings across a broader operational envelope.

## 5. Conclusion

The Taguchi technique, as part of the DOE framework, was employed in this optimization study to explore the influence of critical design parameters of dimples integrated into the BWB airframe. Dimples were proposed as a passive flow control method aimed at improving the aerodynamic performance of lifting surfaces. The trade studies were conducted using high-fidelity CFD simulations, with the goal of exploring the design space efficiently in terms of resources, while preserving the accuracy of the analysis. This approach was intended to extract optimization guidelines and provide a framework that can be applied to similar studies focused on passive flow control techniques.

The dimple location (*x/c*), indentation depth (*d*_*id*_), diameter (*D*_*d*_), spacing between dimples (*P*_*d*_) and rows of dimple (*r*_*d*_) were considered as the design variables, while the performance criteria were the evaluation of *C*_*D*_ and *L/D* ratio. The primary conclusions derived from this optimization study are summarized as follows:

- The Taguchi method significantly reduced the number of necessary numerical simulations by approximately 90% compared to a full factorial “experiment”, thereby maintaining high accuracy in the simulations while minimizing resource consumption.
- ANOVA was performed on SNRs for each performance metric to determine the impact of each design variable. The outcomes indicated that the parameters affecting the δ*C*_*D*_ and δ*L/D* were primarily influenced by *D*_*d*_, while *d*_*id*_ had the least effect.
- The optimal combination of design parameters for maximizing drag reduction was identified as A_1_B_1_C_1_D_3_E_3_, with corresponding values of *x/c* =  SS at 0.85*c*_*k*_, *d*_*id*_ =  0.025*D*_*d*_, *D*_*d*_ =  0.01*c*_*k*_, *P*_*d*_ =  3.5*D*_*d*_ and *r*_*d*_ =  4. *D*_*d*_ (C) was the most influential factor for δ*C*_*D*_, with a contribution factor of 35.19%.
- Similarly, the optimal combination for maximizing the *L/D* ratio was also A_1_B_1_C_1_D_3_E_3_, with the same level values as for drag reduction. *D*_*d*_ (C) had the most significant effect on the *L/D* ratio, with a contribution of 40%.
- Optimized dimple layouts can significantly enhance the aerodynamic performance of the BWB airframe. However, non-optimized designs may degrade aerodynamic efficiency.

In summary, the CFD optimization study, in conjunction with the DOE method and the Taguchi technique, enabled the identification of design trends and optimization results that would otherwise have required significantly more resources and time. Although this study focused on a specific BWB airframe configuration with dimples, the proposed methodology is adaptable to other passive control device optimizations for various aircraft types, facilitating efficient trade studies.
